# A Multi-Objective Approach for Anti-Osteosarcoma Cancer Agents Discovery through Drug Repurposing

**DOI:** 10.3390/ph13110409

**Published:** 2020-11-22

**Authors:** Alejandro Cabrera-Andrade, Andrés López-Cortés, Gabriela Jaramillo-Koupermann, Humberto González-Díaz, Alejandro Pazos, Cristian R. Munteanu, Yunierkis Pérez-Castillo, Eduardo Tejera

**Affiliations:** 1 Grupo de Bio-Quimioinformática, Universidad de Las Américas, Quito 170125, Ecuador; yunierkis.perez@udla.edu.ec; 2 Carrera de Enfermería, Facultad de Ciencias de la Salud, Universidad de Las Américas, Quito 170125, Ecuador; 3 Department of Computer Science and Information Technologies, Faculty of Computer Science, University of A Coruña, CITIC, Campus Elviña s/n, 15071 A Coruña, Spain; aalc84@gmail.com (A.L.-C.); apazos@udc.es (A.P.); c.munteanu@udc.es (C.R.M.); 4 Centro de Investigación Genética y Genómica, Facultad de Ciencias de la Salud Eugenio Espejo, Universidad UTE, Quito 170129, Ecuador; 5 Latin American Network for Implementation and Validation of Clinical Pharmacogenomics Guidelines (RELIVAF-CYTED), 28029 Madrid, Spain; 6 Laboratorio de Biología Molecular, Subproceso de Anatomía Patológica, Hospital de Especialidades Eugenio Espejo, Quito 170403, Ecuador; gaby_jaramillok@yahoo.com; 7 Department of Organic and Inorganic Chemistry, and Basque Center for Biophysics CSIC-UPV/EHU, University of the Basque Country UPV/EHU, 48940 Leioa, Spain; humberto.gonzalezdiaz@ehu.es; 8 IKERBASQUE, Basque Foundation for Science, 48011 Bilbao, Spain; 9 Biomedical Research Institute of A Coruña (INIBIC), University Hospital Complex of A Coruña (CHUAC), 15006 A Coruña, Spain; 10 Escuela de Ciencias Físicas y Matemáticas, Universidad de Las Américas, Quito 170125, Ecuador; 11 Facultad de Ingeniería y Ciencias Agropecuarias, Universidad de Las Américas, Quito 170125, Ecuador

**Keywords:** osteosarcoma, machine learning, multi-objective model, virtual screening, drug repositioning

## Abstract

Osteosarcoma is the most common type of primary malignant bone tumor. Although nowadays 5-year survival rates can reach up to 60–70%, acute complications and late effects of osteosarcoma therapy are two of the limiting factors in treatments. We developed a multi-objective algorithm for the repurposing of new anti-osteosarcoma drugs, based on the modeling of molecules with described activity for HOS, MG63, SAOS2, and U2OS cell lines in the ChEMBL database. Several predictive models were obtained for each cell line and those with accuracy greater than 0.8 were integrated into a desirability function for the final multi-objective model. An exhaustive exploration of model combinations was carried out to obtain the best multi-objective model in virtual screening. For the top 1% of the screened list, the final model showed a BEDROC = 0.562, EF = 27.6, and AUC = 0.653. The repositioning was performed on 2218 molecules described in DrugBank. Within the top-ranked drugs, we found: temsirolimus, paclitaxel, sirolimus, everolimus, and cabazitaxel, which are antineoplastic drugs described in clinical trials for cancer in general. Interestingly, we found several broad-spectrum antibiotics and antiretroviral agents. This powerful model predicts several drugs that should be studied in depth to find new chemotherapy regimens and to propose new strategies for osteosarcoma treatment.

## 1. Introduction

Osteosarcoma (OS) is the most common primary bone tumor in children, adolescents and young adults, representing approximately 3.5% of all childhood cancers and 56% of malignant bone tumors in children. Its incidence rate ranges between 1 and 5 cases per million people and it is usually diagnosed in patients who are 10 to 19 years old. OS follows a bimodal distribution, with an initial peak in late adolescence and young adulthood and a second peak in old age [1].

The management of patients diagnosed with OS has not changed in recent decades. Current systemic OS first-line therapy includes cycles of cisplatin, doxorubicin, and high-dose methotrexate (MAP). Second-line therapy can integrate some tyrosine kinase inhibitors such as sorafenib and everolimus, plus antineoplastic agents like etoposide, topotecan and cyclophosphamide [2]. Neoadjuvant chemotherapy is generally administered for a period of 10 weeks, followed by the surgical resection of the compromised tumor area and radiotherapy. If 90% or more of the tumor area shows necrosis, additional cycles of postoperative therapy are applied to reject micrometastasis [3,4].

The prognosis of this disease is highly variable, possibly due to its high rate of tumor mutations, which leads to widespread dysregulation in cell signaling pathways and genomic instability [5]. Patients with localized disease show a 5-year survival rate of 65 to 70%, while for those who develop metastases the rate drops to 19–30% [6]. These metastatic events involve the lung parenchyma in 75% of the cases and distant skeletal sites [7,8], hindering treatment efficacy [9]. In this scenario, current therapy shows little response sensitivity and the survival rate decreases considerably. 

Despite current chemotherapy regimens being the most effective strategy for OS treatment, patients’ sensitivity to these agents regarding the toxic side-effects and antitumor effects varies considerably [10,11]. Several clinical trials have developed experimental designs to improve survival rates by testing dose intensification, and also adding or combining various chemotherapeutic agents [12]. There is a dose effect on treatment response, but several studies have shown that high-dose chemotherapy may not increase survival rates any more than less toxic moderate doses [13]. Due to the lack of tumor specificity or metastasis events or the complex etiology of these bone tumors, the anti-OS compounds currently used have a narrow therapeutic index and no increase in survival rates have been achieved in the last three decades [14], thus the therapeutic strategies need to be optimized.

The development and validation of novel therapeutic compounds is a time-consuming and labor-intensive process. Drug repositioning, which explores potential novel uses for known molecules based on prediction algorithms has become an effective and innovative approach [15,16,17]. One approach is based on multi-objective computational models where the repositioning process is addressed from a set of potentially desirable solutions. To do this, some computational techniques have been applied and these include Quantitative Structure-Activity Relationship (QSAR) and Ligand-Based Virtual Screening, which aid the identification of hit structures [18,19,20,21]. These QSAR studies are used to perform virtual drug screening that has been integrated into the drug discovery pipeline and could save both time and money, especially in the early phase of drug discovery [22,23]. 

In this sense, the application of these models is of high interest to researchers specializing in cancer. Several studies have focused on the description of new therapeutic agents, especially for treating carcinomas [24,25,26,27,28,29,30,31,32]. However, very few have concentrated on tumors of mesenchymal origin [33,34]. Thus, we developed a multi-objective model for the prediction of drugs with potential biological activity towards OS, one of the most prevalent cancers in pediatric populations where current chemotherapy treatments have not varied in the last decades.

## 2. Results

### 2.1. Datasets and Molecular Descriptors

The ChEMBL database reports a total of 1250 compounds with biological activity for HOS, MG63, U2OS, and SAOS2 cell lines. Of these, 1036 shows complete information on their biological activities evaluated by IC_50_, GI_50_, and C_50_ assays (Table S1). Before constructing the prediction algorithms for each cell line, we inspected all those compounds reported in the DrugBank and separated them from this list for later use in virtual screening (VS).

**Figure 1:**
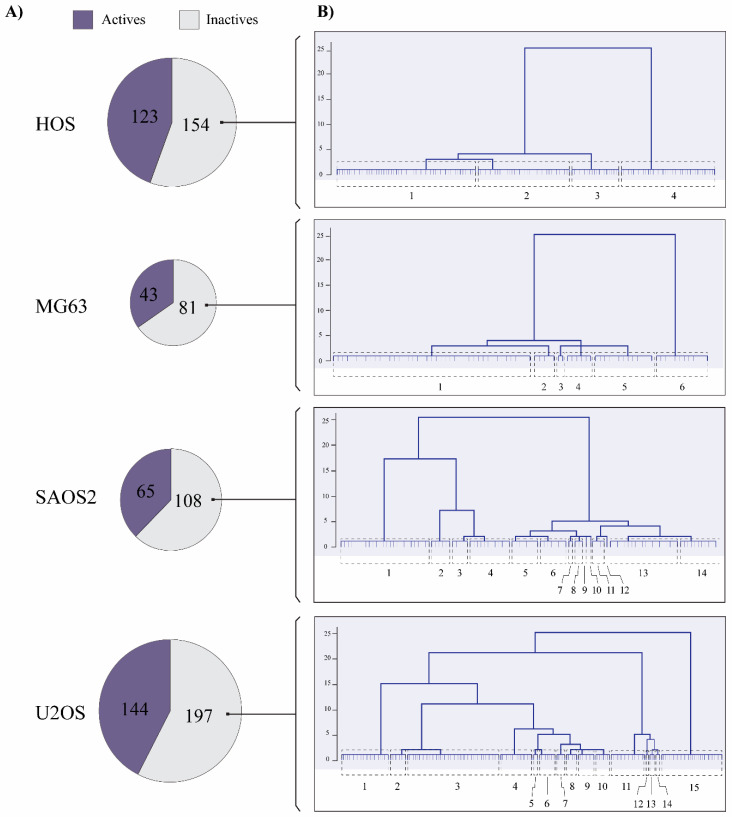
The chemical diversity of inactive compounds in OS cell lines. (**A**) Compounds with biological activity reported in ChEMBL for OS cell lines. (**B**) Dendrograms calculated for inactive compounds in the HOS, MG63, SAOS2 and U2OS cell lines.

Of the 1036 compounds, 28 drugs are reported in DrugBank for the HOS cell line, 30 for MG63, 31 for SAOS2, and 32 for U2OS. In this way, we removed these 121 drugs from the 1036, and the prediction models were built on the remaining 915 compounds. Thus, the calculation of the molecular descriptors (MDs) was performed on 277 compounds described for HOS, 124 for MG63, 173 for U2OS and 341 for SAOS2 (Figure 1A) and we obtained 500 ISIDA variables for each cell line (Table S2).

Since inactive compounds are described in greater numbers than active ones in all cell lines, we evaluated the chemical diversity in the inactive series by applying a hierarchical clustering. Thus, we calculated the degree of similarity in the inactive compounds to balance the data through stratified random sampling instead of a solely random partitioning. As a result, hierarchical representations were generated in which the clusters at each level of the hierarchy were created by merging clusters at the next level down [35]. In our case, we chose a strict cut-off to show all the possible groups and to make sure we had a wide representation of the chemical space within the data in the inactive series. From this, we identified four clusters in the HOS cell line, six in MG63, 14 in SAOS2 and 15 in U2OS (Figure 1B). 

Therefore, we separated 24 inactive compounds for all cell lines (24 in each) reported in the DrugBank, as mentioned above, and then 7, 14, 19 and 29 compounds from each list. The balanced datasets resulted in 246 molecules for HOS, 86 for MG63, 130 for SAOS2, and 288 for U2OS, with a ratio of 1:1 between active and inactive compounds. 

### 2.2. Construction of Models

The prediction algorithms used were: support vector machine (SVM), random forest (RF), neural networks (NN), decision tree (DTREE), k-Nearest Neighbors (KNN), and a scalable end-to-end tree boosting system (XGBoost) [36]. 

**Figure 2:**
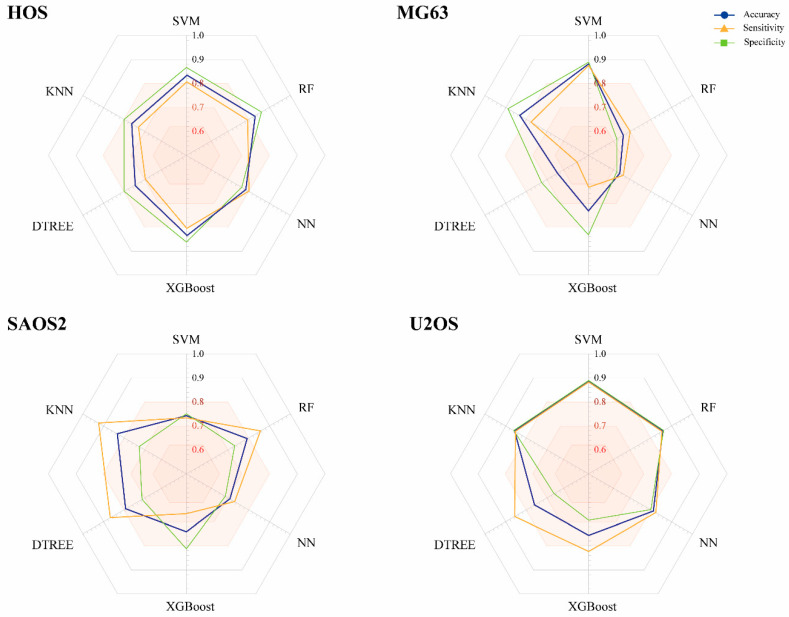
Performance of machine learning models constructed from compounds with biological activity for the HOS, MG63, SAOS2 and U2OS cell lines. Accuracy, sensitivity and specificity values correspond to the external data.

As seen in Figure 2, each of the six trained models demonstrated different performance metrics on the external data. HOS had similar achievements in the six learning techniques, but only the SVM, RF and XGBoost models showed optimal accuracy (AC) for subsequent assembly (0.836, 0.828 and 0.833 respectively). For MG63, the best strategies were SVM (0.882) and KNN (0.833). Prediction values when using RF, NN, XGBoost DTREE were less than 80%, and the true positive rate was lower than 0.7.

SAOS2 modeling resulted in only one algorithm with AC greater than 0.8, namely KNN (0.833). Lastly, the prediction models for U2OS with ACs > 0.8 were SVM, RF, NN, and KNN (0.886, 0.857, 0.812, and 0.857 respectively), all of whose SN and SP rates were higher than 0.8. XGBoost and DTREE were not taken into account for the assembly.

It is interesting to note that the feature selection by genetic algorithm (GA) reduced from 500 to 19–101, depending on each dataset. This strategy allowed us to generate the models described above and obtain the desired performance measurements for the final model (Table S3). 

### 2.3. Multi-Objective Model Assessment and Virtual Screening

The AC, SN and specificity (SP) evaluated the performance of a model based on its training and data described as external, but these metrics do not always describe a desirable recovery rate at the time of performing screening for drug repositioning [37]. Therefore, the multi-objective model was evaluated in a virtual screening setting, where we mainly took into account the Area Under the Accumulation Curve (AUC), the Boltzmann-Enhanced Discrimination of ROC (BEDROC) and recovery efficiency (EF) at 1% of the screened list. The VS was developed on a dataset of 772 compounds. Of these, 14 corresponded to drugs used for previously described OS treatment, 653 were decoy molecules calculated from these 14 drugs, and 105 compounds were described as inactive and removed during the data balancing process.

We used all base-models with an AC greater than 0.8 in previously described external validation, and tested all possible combinations. Based on the VS results, the best multi-objective model was made up of the desirability values of the algorithms HOS-SVM, HOS-RF, MG63-SVM, SAOS2-KNN, U2OS-NN, and U2OS-KNN.

**Figure 3:**
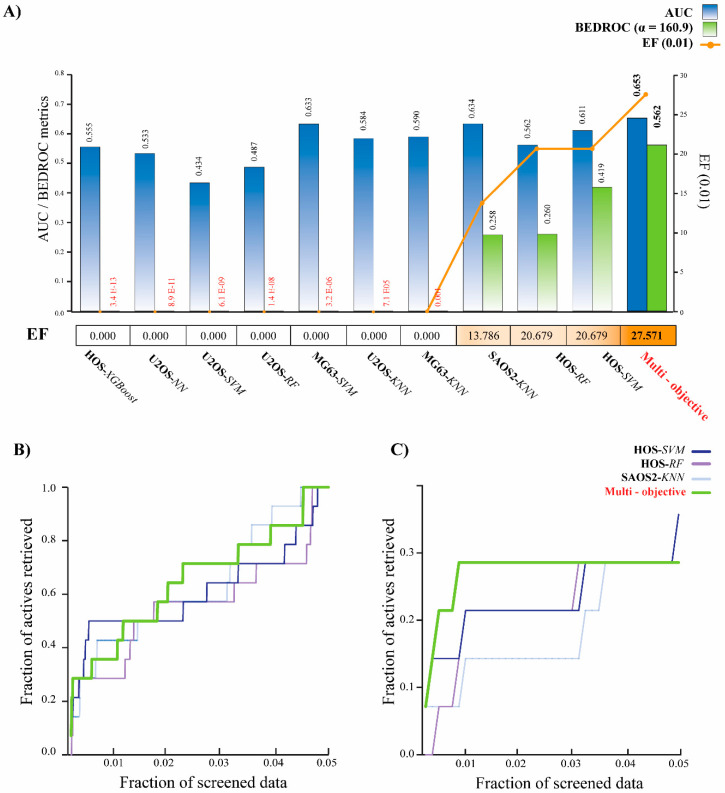
Results of the performance of base models and multi-objective models for the VS. (**A**) Comparison of AUC values (black bars) and BEDROC with a = 160.9 of base models and the multi-objective algorithm. (**B**) Accumulative curves for the four top-performing VS protocols. The comparison includes the best 3 simple models and the multi-objective algorithm. Results are presented for the whole screening, and (**C**) for the top 5% of screened data.

As seen in Figure 3A, our strategy generated a prediction method with better early recognition rates than the individual models. BEDROC is a metric that assigns more weight to early ranked molecules than late ranked molecules, therefore the initial enrichment was weighted. This enrichment was higher in our multi-objective model (BEDROC = 0.562) when calculated with an α = 160.9. This means that our algorithm turned out to be the best strategy for recognizing “active” anti-sarcoma molecules in 1% of the list of therapeutic drugs for OS. Likewise, the EF value was higher in our algorithm when analyzing the recovery rate at 0.01. The EF values calculated for a recovery efficiency of 1% in the base models HOS-RF, SAOS2-KNN, and HOS-SVM were 20.68, 13.8, and 20.68, respectively, while in our method they reached 27.57. This indicates that with our protocol, it is possible to retrieve almost 27 times more the number of multi-targeted compounds in the first 1% of the ranked list than what is expected from a uniform distribution of the active ones in the virtual screening database.

When analyzing the active retrieved fractions of all the models (Figure 3B), one notices that all protocols have similar recovery rates within 30% of the data screened. However, a closer inspection of the screened data shows that the multi-objective has the highest recovery rate of anti-OS compounds at 1% (or less) of the data screened (Figure 3C). This algorithm recognized four compounds within the first six positions. These retrieve rates suggest that a strategy made up of several methods is capable of predicting those molecules described as active within 1% of a screened list. In a drug repositioning scenario, this is important since the compounds ranked in the first positions have a high probability of presenting biological activity in vitro. Using our prediction algorithm, a prediction rate of 59.9% would be expected in 1% of screening for drugs with anti-OS activity.

### 2.4. Analysis of Repurposed Drugs

**Figure 4:**
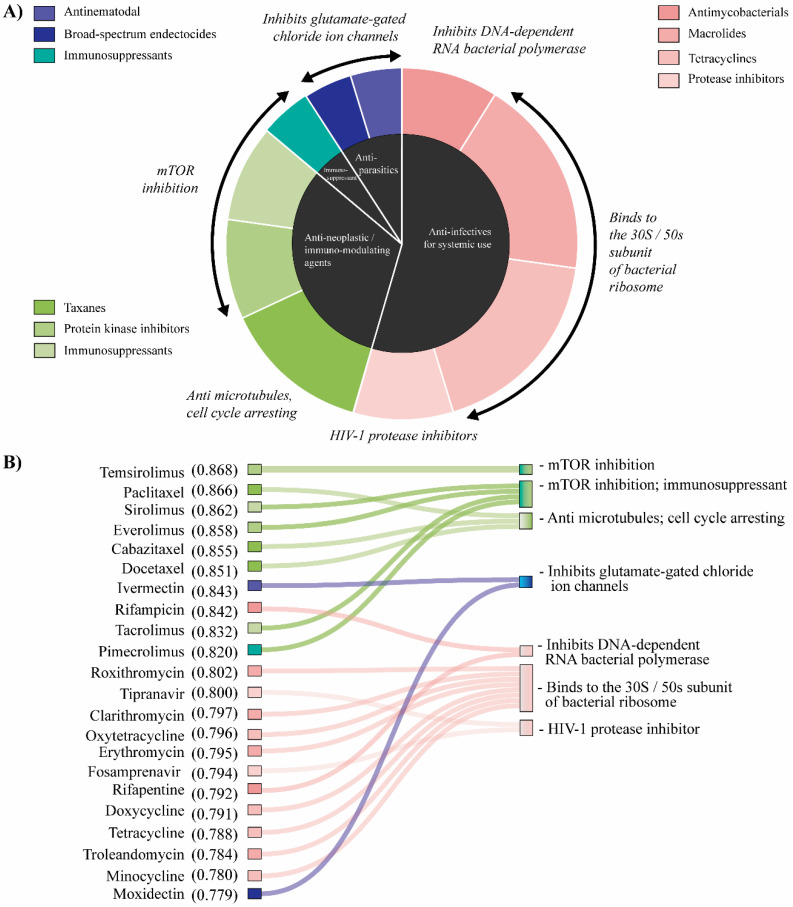
Repositioned drugs for OS treatment. (**A**) The central pie chart (black) shows the distribution of the 4 main drug classes repositioned at the first 1% of the screened list, whereas the outer pie chart shows the groups they represent. Each color represents a specific group obtained from the Anatomical Therapeutic Chemical (ATC) classification system. The action mechanisms of the screened drugs are also included in italics. (**B**) Correlation between the top-ranked drugs using the multi-objective model and its action mechanism. Listed are the first 22 positions (1%) of the 2218 DrugBank compounds screened. Drugs and their desirability values obtained using the prediction algorithm are described in the left column, while their action mechanism is in the right column. The colors represent the drug groups described in the graph above.

The screening weighted four principal drug classes in the first 1% of the screened list: anti-infectives for systemic use (antimycobacterials, macrolides, protease inhibitors and tetracyclines; which represent 55%); antineoplastic/immunomodulating agents (immunosuppressants, protein kinase inhibitors and taxanes; 32%); dermatological/immunosuppressant (agents for dermatitis, excluding corticosteroids; 4%); and antiparasitics (an antinematodal agent and a broad-spectrum endectocide; 9%). The first two groups represent more than 85% of all repositioned drugs (Figure 4A). All the desirability values for each repositioned drug are detailed in Table S4.

The action mechanism of antineoplastic and immunomodulating agent mainly inhibits the mTOR pathway and microtubules polymerization. Among these, temsirolimus, paclitaxel, sirolimus (rapamicine), everolimus, cabazitaxel and docetaxel are ranked at the top. On the other hand, broad-spectrum antibacterials described as drugs that bind to the 30S/50s subunit of bacterial ribosome, HIV-1 protease inhibitors and antimycobacterials, which inhibit DNA-dependent RNA bacterial polymerase, were weighted in the screening. We also found two molecules used for the treatment of HIV, described as inhibitors of HIV-1 protease (tipranavir and fosamprenavir) (Figure 4B). It is interesting to note that several repositioned drugs have been found within clinical trials for cancer patients. Out of the antineoplastic and immunomodulating agents, only cabazitaxel has not yet been studied in trials related to bone sarcomas. Moreover, broad-spectrum antibacterial compounds such as clarithromycin, erythromycin, doxycycline and tetracycline are top-ranked drugs that are registered in clinical trials for carcinomas.

## 3. Discussion

Several reports have shown that multi-objective models have a better prediction rate during screening time since they approach the problem with a particular perspective from a set of potentially desirable solutions [18,21,38,39]. In our case, each of these possible desirable solutions was made up of each algorithm constructed from the described compounds with activity for the OS cell lines HOS, MG63, SAOS2 and U2OS. One of the major outcomes of this study is the improvement of the AUC and BEDROC values obtained in the VS, especially the EF of the multi-objective model when comparing with the performance of the base models (Figure 3A). This suggests that our algorithm improves the recognition rate of molecules described as therapeutic for OS treatment, especially within 1% of the data screened (Figure 3B). Specifically, the EF obtained indicates that it is possible to retrieve in the first 1% of the ranked list almost 27 times more multi-targeted compounds than what is expected from a uniform distribution of the active ones in the virtual screening database, something that is not obtained from the algorithms generated by each cell line.

Drug repositioning is an effective strategy for finding novel drug-disease relationships for existing molecules. The development of these strategies has gained considerable interest in recent years compared with de novo drug discovery pipeline, which demands more research time and experimental hours in the case of new drug development, and requires a greater financial investment. On the other hand, the use of already proven drugs is a highly efficient, low-cost and low-risk strategy since screening is carried out on molecules that have passed all clinical safety tests at Phase I, Phase II, and Phase III [40,41]. We used our multi-objective approach in order to propose new agents with chemotherapeutic activity for osteosarcoma treatment. Given the high recovery rate of active compounds obtained in our model (EF 0.01 = 27,571), we considered the first 22 highest-ranking compounds belonging to 1% of the 2218 approved FDA drugs reported in the DrugBank.

Of these 22 drugs, 13 (59.1%) are enrolled in clinical trials for cancer patients (reviewed at https://clinicaltrials.gov/) (Table S5): temsirolimus, paclitaxel, sirolimus/rapamycin, everolimus, cabazitaxel, docetaxel, rifampicin, tacrolimus, clarithromycin, erylinethromycin, doxycycline, tetracycline and minocycin. Interestingly, only five of these drugs are included in trials of patients with OS: temsirolimus, paclitaxel, sirolimus/rapamicin, everolimus, and docetaxel. The remaining 10 drugs (ivermectin, pimecrolimus, roxithromycin, tipranavir, oxytetracycline, fosamprenavir, rifapentine, troleandomycin and moxidectin) are not registered in any clinical trial for cancer patients, however, their action mechanisms are similar to various chemotherapeutic agents used in oncology practice.

Cancer cells are characterized by unregulated proliferation, which leads to cellular undifferentiation and disruption on the function of tissues. Cell proliferation can be caused by a checkpoint failure in cell cycle or a disruption in the cell death pathway. In this sense, any agent that affects the metabolism of cancer cells by reducing or inhibiting cell proliferation and promotes apoptosis is a potential target for cancer treatment [42]. Several agents used as first-line treatment for OS, such as methotrexate, doxorubicin, etoposide, cisplatin and ifosfamide, induce a disruption in these cellular functions, either by interrupting nucleotide synthesis, by DNA synthesis by inhibiting topoisomerase II, or by binding to double-strand DNA to promote apoptosis. On the other hand, several second-line drugs act on mTOR, a pathway considered pathogenic within the development and progression of OS [43,44,45], and on the formation of microtubules, inhibiting the progression from the G1 to the S phase of the cell cycle. In the top six positions of our screening, we found chemotherapeutic drugs described as therapeutic agents for various types of cancer (temsirolimus, paclitaxel, sirolimus, everolimus, cabazitaxel and docetaxel). Indeed, these compounds belong to one of the four principal drug classes found in our repositioning called antineoplastic and immune-modulatory agents (Figure 4A). Their action mechanism resembles those previously described as second-line drugs, which mainly inhibit mTOR and interfere with the microtubule depolymerization (Figure 4B). Interestingly, cabazitaxel is the only one of these six top-ranked compounds that is not reported in clinical trials of OS patients. This molecule is a semi-synthetic derivative of a natural taxoid that considerably increases overall survival versus mitoxantrone after prior docetaxel treatment in patients with metastatic castration-resistant prostate cancer [46,47,48]. Cabazitaxel induces cell cycle arresting by interacting with the microtubule depolymerization by what is defined as a microtubule destabilizing agent. These types of agents show high antineoplastic activity and have been reported in previous studies into drug repositioning [49]. Although they commonly used in pediatric oncology [50], the microtubule-stabilizing taxanes are not often used to treat childhood cancers due to limited activity, even if safety is observed [51]. In this sense, cabazitaxel can be an important therapeutic agent for the treatment of OS, especially in patients who can progress onto it after docetaxel.

It is interesting to note that 54.5% of the total predicted compounds (12 out of 22) are classified as anti-infectives for systemic use. More specifically, taking into account the Anatomical Therapeutic Chemical (ATC) classification system, our protocol weighted several macrolides (roxithromycin, clarithromycin, erythromycin and troleandomycin), tetracyclines (oxytetracycline, doxycycline, tetracycline and minocycline), protease inhibitors (tipranavir and fosamprenavir) and antimycobacterial (rifampicin and rifapentine) as possible anti-OS agents (Figure 4B). On the one hand, prior studies into cancer therapy have noted the importance of macrolide and tetracycline compounds in cancer treatment [52,53]. Some authors have suggested that these groups of compounds inhibit the action of matrix metalloproteinases (MMPs) in order to reduce the degree of tumor invasion and metastases [54]. Others have observed that these drugs act on mitochondrial biogenesis [55,56], disrupting this process and thus increasing the effectiveness of chemotherapy or radiotherapy on tumor cells. On the other hand, the therapeutic action of HIV-protease inhibitors for the treatment of cancer has been reported. Although these molecules are not expected to cross-react with human peptides, preclinical data suggest that their antitumor activity may be linked in part to the inhibition of endopeptidases, such as metalloproteases and proteasomes [57]. Of our repositioned drugs, clarithromycin, erythromycin and doxycycline are currently under study as possible therapeutic agents for leukemia, colorectal, prostate and lung cancer, among others [58,59,60,61], and are involved in clinical trials of cancer patients (Table S5). Based on our findings, these agents could demonstrate antitumor activity in bone tumors.

These results may be promising for future preclinical and clinical studies. The lack of therapeutic options for OS should be the basis of searches for new agents as potential treatments. The discovery of molecular targets in OS will be part of the development of new molecules that could give these patients more options.

## 4. Materials and Methods

### 4.1. Preprocessing Datasets and Molecular Descriptors

Prediction models were developed from compounds described in the Chemical database (Version 25) of the European Molecular Biology Laboratory (ChEMBL) [62,63] with biological activity against the OS cell lines HOS (ChEMBL614736), MG63 (ChEMBL614347), SAOS (ChEMBL614894) and U2OS (ChEMBL615023). We considered all standard values evaluated by IC_50_ (half-maximal inhibitory concentration), GI_50_ (percentage of cell grow inhibition at a fixed concentration), and EC_50_ (a concentration that inhibited half the cell culture growth), and from these scores, we defined a class for each compound.

Compounds with standard values > 10 µM were classified as inactive (0), and those with values < 10 µM as active (1). In those drugs where two or more assays are reported, and the standard values classify these compounds in different classes, the final criteria were assigned by most of the set of classes obtained. If more than 75% of the tests obtained classify a compound in the same class, this drug was included in the study, otherwise it was rejected. On the other hand, compounds that did not show information about their biological activity, inconclusive data about their activity, or incomplete information regarding ChEMBL ID or canonical SMILES were removed from the analysis.

We used the ChemAxon’s JChem for Excel (18.8.0.253) [64] software to code the chemical structures in SMILES format. This information was converted to SD files (SDFs) and the structure of each compound was standardized using ChemAxon’s Standardizer [65]. Explicit hydrogen atoms were removed. Then we normalized specific chemotypes, such as nitro to one unique representation, the rings aromatization, the curation of tautomeric forms, the striping of salts and small fragments. Furthermore, all duplicate structures were identified using the EdiSDF tool within the ISIDA/QSPR package and subsequently withdrawn from the list [66].

Two-dimensional molecular descriptors were computed with ISIDA Fragmentor 2017 [67,68]. The types of descriptors calculated were: Sequences of atoms and bonds; Atom-centered fragments based on sequences of atoms and bonds; Atom-centered fragments based on sequences of atoms and bonds of fixed length; and Triplets. For these calculations, the minimum and maximum length of fragments as sequences were set to 2 and 8, respectively. Molecular descriptors were calculated separately for each dataset.

The computed descriptors for each dataset were first filtered to remove those present in less than 1% of the compounds. Next, the Minimal Redundancy Maximal Relevance (mRMR) algorithm [69] was employed to keep the top 500 features in each dataset. For mRMR, the Mutual Information Quotient (MIQ) score was used as a features-ranking metric. This subset of 500 selected molecular descriptors was employed for QSAR modeling.

### 4.2. Machine Learning Models and Quality Evaluation

In each cell line under study, the amount of active and inactive compounds varied considerably. This unbalance in the dataset is not desirable for modeling. In order to balance the classes, the following procedure was executed in all cell lines. (1) Using the previously computed molecular descriptors, we carried out a hierarchical clustering. We applied the interval measure, the Euclidean distance and Ward’s method for clustering, both in active and inactive compounds for each cell line. The IBM SPSS Statistics software v.25 (IBM Corp., Armonk, NY, USA) was employed to generate dendrograms and define all the clusters within the data. (2) Once the number of clusters had been defined, we continued with a random stratify extraction of the same amount of compound in both classes. This procedure had previously been used by other authors in order to obtain a balanced data representative of the chemical diversity space [70]. The training set consisted of 75% of randomly chosen compounds from the balanced dataset and the remaining percentage was utilized as external data. The external dataset was used to evaluate the model performance metrics.

To obtain each model, we applied genetic algorithms as feature selection by considering an initial population of 50 chromosomes and 30 generations. For validation of the fitness function in GA, we performed a cross-validation strategy using the average balanced classification rate (BRC) across 100 random splits (bootstrap sampling). This means that in each generation, 100 models were evaluated and the average AC was extracted. The models used together with genetic algorithm were: the support vector machine, random forest, neural networks, decision tree, k-Nearest Neighbors, and a scalable end-to-end tree boosting system [36]. The SVM kernel was fixed to RBF. For performance metrics of models, we calculated the total accuracy (AC), sensitivity (SN), specificity (SP) and the balanced classification rate (BCR) as follows:

(1) $$AC=\frac{Number\;of\;correctly\;classified\;compounds}{Total\;number\;of\;compounds}$$

(2) $$SN=\frac{Number\;of\;correctly\;classified\;active\;compounds}{Total\;number\;active\;compounds}$$

(3) $$SP=\frac{Number\;of\;correctly\;classified\;inactive\;compounds}{Total\;number\;inactive\;compounds}$$

(4) $$BCR=\frac{SN+SP}{2}\times \left(1-|SN-SP|\right)$$

### 4.3. Multi-Objective Model Assembly and Virtual Screening

The construction of the multi-objective model was performed by computing the global desirability as:

(5) $$D_{1}=\left(d\left(y_{1}\right)d\left(y_{2}\right),\;\ldots ,\;d(y_{k}\right){)}^{1/k}$$

where $y_{k}$ corresponds with the desirability scores of each cell line (*k* = 1, …, 4). For each of the cell lines, several possible models are available. The resulting prediction of each model for a given compound resulted in a score linked to the class membership- either a prediction score for an active class, and/or score for an inactive class. In all cases, we established the score for which the compound was active against the cell line. However, the calculated class membership for some machine learning algorithm occurred in the range of positives and negatives, where active compounds showed positive values and vice versa. For all the cases, the geometrical mean of all scores of the compound to be active in a particular cell line (or to be 0–1, normalized transformation) was used as a desirability score for each model ($y_{k}$). Since there are several possible combinations, we performed an exhaustive exploration to obtain the best possible model. Hence, we explored the combination of all possible models in the computation of each $d\left(y_{k}\right)$ and consequently D_1_ in order to obtain the best performance in early recognition metrics for virtual screening.

For VS, we developed a dataset with those antitumor compounds used in the current management of osteosarcoma, not included in either the training or external sets for any cell line and with compounds validated in clinical studies for OS, published on the US government’s Clinical Trials website (https://clinicaltrials.gov/). As first- and second-line therapy drugs, we included [4,12,71,72]: doxorubicin (ChEMBL53463), methotrexate (ChEMBL34259), ifosfamide (ChEMBL1024), etoposide (ChEMBL44657), sorafenib (ChEMBL1336), cyclophosphamide (ChEMBL88), docetaxel (ChEMBL92), gemcitabine (ChEMBL888), dactinomycin (ChEMBL1554) and vincristine (ChEMBL90555). Additionally, we incorporated as validated drugs in clinical trials: temsirolimus (ChEMBL1201182) [73,74], ridaforolimus (ChEMBL2103839) [75,76], sirolimus (ChEMBL413) [77] and pazopanib (ChEMBL477772) [78,79].

As inactive compounds for screening, we considered those molecules withdrawn in the data balancing process (previously described, thus not employed for model training and selection), and common ChEMBL compounds for the four cell lines that showed no biological activity (standard values > 10 µM). Additionally, we generated Decoy molecules based on the selected active compounds by employing the DUD-E server 5 [80]. We incorporated around 50 inactive molecules for each active compound, which is the proportion used in the DUD-E database that is widely employed to validate virtual screening workflows [80].

The performance of our models within this VS scenario was evaluated by computing the AUAC, BEDROC and EF [81,82]:

(6) $$AUC=1-\frac{1}{n}\sum_{i=1}^{n}x_{i}$$

(7) $$EF=\frac{\sum_{i=1}^{n}\delta_{i}}{\chi^{n}},{\mathrm{where}\;\delta }_{i}=\left\{\begin{array}{c} 1\;r_{i}\leq \chi^{N}\\ 0\;r_{i}>\chi^{N} \end{array}\right\}$$

(8) $$BEDROC=\frac{RIE-RIE_{min}}{RIE_{max}-RIE_{min}},\mathrm{with}\;RIE_{min}=\frac{1-e^{\alpha R_{a}}}{R_{a}\left(1-e^{\alpha }\right)},RIE_{max}=\frac{1-e^{-\alpha R_{a}}}{R_{a}\left(1-e{-}^{\alpha }\right)}\;\mathrm{and}\;RIE=\frac{\frac{1}{n}\sum_{i=1}^{n}e^{-\alpha x_{i}}}{\frac{1}{N}\left(\frac{1-e^{-\alpha }}{e^{\alpha /N}-1}\right)}$$

In the above equations, *n* represents the number of active compounds, *N* the total number of compounds in the dataset, *x_i_* the relative ranking of active compound *i* in the ranked list, *χ* the fraction of data for which EF will be computed, *R_a_* the rate of active compounds in the dataset (*n*/*N*), and *α* is the α parameter which ensures that active compounds ranked at the beginning of the ordered list result in higher weights than those at the tail. The *α* parameter is computed using the following equation:

(9) $$\theta \left(1-e^{-\alpha }\right)-1+e^{-\alpha z}=0$$

where *z* represents the fraction of the ranked list at which enrichment is important and *θ* is the expected contribution of the enrichment at this *z*% fraction to the overall enrichment.

## 5. Conclusions

In conclusion, this study presents a multi-objective prediction algorithm developed from compounds described with biological activity for the osteosarcoma cell lines HOS, MG63, SAOS2 and U2OS. The performance of this multi-objective model considerably improves the recognition rate in a virtual screening scenario, developed on drugs used as first- and second-line treatment for OS. Specifically, a high level of performance was observed for the recognition of molecules with biological activity within 1%. Using this ML algorithm on 2218 compounds described in the DrugBank, we found several antineoplastic agents currently being studied in clinical trials for the treatment of OS. Interestingly, Cabazitaxel is a compound with chemotherapeutic activity that is being studied in several clinical trials for different types of carcinomas and not in sarcomas, therefore it can be taken into account for clinical validations in patients with OS. Furthermore, several broad-spectrum antibiotics, for instance clarithromycin, erythromycin and doxycycline, were top-ranked drugs in our screening. These compounds have already been studied in various types of carcinomas, so they comprise an interesting group of drugs for developing therapeutic validation studies in bone cancers. One of the main limitations was the lack of experimental validation of the drugs proposed for repositioning because this was an initial study. Although it is true that several compounds have already been studied in clinical trials, the validation process of their biological activities on bone tumor cells is an indispensable step in order to proceed with a validation strategy in patients, hence this will be the next procedure in further studies.

## Supplementary Materials

The following are available online at https://www.mdpi.com/1424-8247/13/11/409/s1. Table S1. ChEMBL dataset of compounds with biological activity for HOS, MG63, U2OS and SAOS2 cell lines. Table S2. ISIDA Molecular descriptors calculated for each cell line’s dataset. Table S3. Performance on machine learning models for HOS, MG63, SAOS2 and U2OS cell lines. Table S4. Ranking and desirability values of drugs repositioned by the multi-objective model. Table S5. Clinical trials reported for the 22 top-ranked compounds repositioned by the multi-objective model.
